# New Appliances and Things Medical

**Published:** 1897-01-09

**Authors:** 


### NEW APPLIANCES AND THINGS MEDICAL.

[We shall be glad to receive, at our Office, 28 & 29, Southampton Street, Strand, London, W.C., from the manufacturers, specimens of all new preparations and appliances which may be brought out from time to time.]

### DATE VINEGAR.

(Victoria Date Company, Belvedere Road, Lambeth, S.E.)

A sample of this new vinegar has been forwarded us for examination. For table use we find it delicate and pleasant in flavour, reminding one of the best examples of wine vinegar, and infinitely superior to many vinegars on the market, which often contain pyroligneous as well as added mineral acids. Although pleasantly pungent, it does not possess the crude acidity of the inferior qualities of acetic acid. It is claimed for it that it is made from the acetous fermentation of date juice, and to be perfectly pure, and as far as our examination of it has gone, we regard these statements as correct, and date vinegar as a distinct improvement, both on the usual malt and vinous varieties.

